# Wind-tunnel tests of synthetic jet control effects on airfoil flow separation

**DOI:** 10.1038/s41598-022-19642-2

**Published:** 2022-12-20

**Authors:** Guoqing Zhao, Qijun Zhao, Xi Chen

**Affiliations:** National Key Laboratory of Rotorcraft Aeromechanics, College of Aerospace Engineering, Nanjing University of Aeronautics and Astronautics, Nanjing, 210016 China

**Keywords:** Aerospace engineering, Fluid dynamics

## Abstract

To find out the comprehensive principles of synthetic jet control effects on airfoil stall characteristics, fundamental contrastive wind-tunnel tests were conducted systematically. By using six-component balance, Particle Image Velocimetry (PIV) technology and boundary layer probe, the measurements of model aerodynamic forces, the whole velocity field over airfoil and velocity profiles in the boundary layer were conducted, respectively. Based upon the experimentally parametric analyses of synthetic jet control on airfoil, it is concluded that the incline angle of synthetic jet has an important impact on both the flow in boundary layer and aerodynamic forces of airfoil. A tangential jet inject energy and accelerate the velocity of flow in inner boundary layer thus has a better control effects on delay stall of airfoil when the momentum coefficient of jet is relatively large, and the normal jet helps to enlarge the thickness of boundary layer which is proved to have better control effects on flow separation control and improving the aerodynamic characteristics of airfoil when the momentum coefficient of a jet is relatively small.

## Introduction

The pitch of retreating blade of rotor is commonly larger than that of advancing blade, especially at high forward speed and large maneuvering flight status of helicopter, as a result,flow separation is easy to occur on retreating blade of rotor at these flight states^1^. Flow separation induced by the leading-edge vortex over the airfoil under high angle of attack may lead to the loss of the lift, the divergence of drag and pitching moment, and further decrease the aerodynamic performance of rotor and induce vibration of blade. Therefore, the delay even suppression of airfoil flow separation and stall is one the focus of helicopter aerodynamic. As an emerging method, active flow control (AFC) for airfoil flow separation and stall has been developed rapidly, among which, synthetic jets can transfer momentum to the surrounded flow and enhance the mixing of the boundary laryer without net mass injection across the flow boundary, thus, compared with the injected jet, it is more suitable for the flow control of airfoil^2^.

Synthetic jet actuator (SJA) was firstly developed by Smith and Glezer in 1994^3^, and then the flow charateristics of synthetic jet were experimentally and numerically investigated^4,5^. Glezer et al. pointed out that the interaction domain between a synthetic jet and a cross flow induce an “apparent” modification of the flow boundary and alter the local pressure and vorticity distributions, and these attributes may be exploited to modify or control the evolution of wall-bounded and free-shear flows on scales that are one to two orders of magnitude larger than the characteristic length scale of the jets themselves^6^. Manikandan et al. further carride out the PIV tests on interactions betweent the sythetic jet and the cross flow^7^, the results indicated that the interaction caused by the synthetic jet was limited to the viscous sublayer and log layer and never reached beyond 60% of the boundary-layer thickness from the surface to interact with or transport energy from the mean flow. Besides, the time evolution of the synthetic jet was founded to be completely different between still air and crossflow, which suggested that any performance prediction, such as momentum added to the boundary layer or the penetration of the jet in terms of boundary-layer thickness based on still air conditions may not be applicable in crossflow conditions at all.

In 2014, Buren et al. measured the three-dimensional flow structures of a finite-span synthetic jet by employing the PIV technique^8^. The experimental resulsts illustrated that when the highest velocity was achieved did not mean the highest momentum was generated, and the added momentum might be idealer for flow control than the maximum velocity of synthetic jet. Therefore, geometry effects of jet orifice might have a pronounced influence on the flow field structures and, as a result, alter the effectiveness of the flow control. After that, Buren et al. conducted PIV experiment on investigaions of flow structures associated with a finite-span synthetic jet^9^. The results show that synthetic jet would result in a boundary layer less susceptible to separation in an adverse pressure gradient flow, and the wall-normal, transverse jet induced the most flow unsteadiness near the orifice, and the unsteadiness decreased with an increase in skew angle and a decrease in pitch angle. Additonally, Buren et al. investigated the influences of orifice aspect ratios on interactions of synthetic jet and zero-pressure gradient laminar boundary layer^10^, it was founded that a decrease in aspect ratio resulted in a reduction in the distance between the edgewise vortex structures and the reduction in aspect ratio reduced the virtual blockage of the jet. Furthermore, Buren’s results suggeted that the spacing and interaction of multiple actuators should be taken into because the unsteady and steady features of the flow were characterized three-dimensionally. Chiatto et al. conducted tests on control of continuous water spray by employing synthetic jet^11^, the experimental results showed that the synthetic jet could energize the region downstream of the impact and producing higher droplet velocity, and the effect tended to reduce as the injection pressure of water spray increases.

The investigations on flow structures of synthetic jet in still and cross flow showed that the interaction of a synthetic jet with an external cross flow can displace the local streamlines and induce an apparent or virtual change in the shape of the surface, thus, the synthetic jet had potential utility for flow control applications, specifically control of the performance of aerodynamic surfaces through fluidic modification of their apparent aerodynamic shape^6,12^. Under such a background, active flow control (AFC) for suppressing airfoil stall has been attached much importance and developed rapidly. So far, the novel periodic excitation method by using synthetic jet is demonstrated to be one of the most promising AFC methods on controlling stall characteristics of an airfoil^13–17^.

Hassan et al. carried out preliminary researches for synthetic jet control of airfoil by employing RANS method with B-L turbulance model^18^, and the numerical results showed that synthetic jet could enhance the aerodynamic characteristics of airfoil under stall. Seifert et al. conducted test for synthetic jet control on NACA0015 airfoil^19^, which partly verified the conclusion of Hassan, and the results indicated that a jet placed just upstream of the separation location was effective on controlling the flow separation of an airfoil. In 2003, Lee et al. experimentally investigated the control effects of synthetic jet on controlling the adverse pressure gradient and boundary layer separation^20^. The results showed that when the synthetic jet actuator was excited by the unstable frequency of boundary layer flow, the modulation control effects of synthetic jet on boundary layer flow was significantly enhanced. Dandois et al. performed numerical simulations on flow separation control by synthetic jet over a smooth ramp^21^, and the influences of frequency were studied. The results showed that the separation length is reduced by half for a low excitation frequency F^+^ = 0.5 of synthetic jet, however, the effect of the high frequency forcing at F^+^ = 4 is a significant increase of the separation length, which indicated the importance of excitation frequency. Sahni et al. carried out experimental and numerical investigations on the interaction of a finite-span synthetic jet with a crossflow over a finite wing at a chord-based Reynolds number Re_c_ = 10^5^ and 0° angle of attack^22^. The reslusts show that the larger the blowing ratio (jet velocity to freestream velocity) was, larger spanwise structures and wavelength were obtained, which could help to enhance the control effect of synthetic jet.

Overall, a variety of numerical and experimental investigations had already been carried out to explore the mechanisms of a synthetic jet on improving the maximum lift of airfoil as well as the stall angle^23–26^. There were some consensuses regarding the parametric analyses of jet parameters, such as the influences from jet momentum coefficient, location of jet and excitation frequency. In the investigations of the implications of synthetic jet location, a general good agreement was concluded that the jet adjacent to the separation position enabled the jet vortex penetrate into large separated flow state more efficiency^27^, and thus it reduced the substantial size of the separation vortex and delay the stall of an airfoil^28,29^. Further, as another influential parameter, the momentum coefficient of the synthetic jet was demonstrated to be proportional to the growth of aerodynamic characteristics of an airfoil in the previous studies^30–32^, and the control effects tended to be stabilized with momentum coefficient exceeding a critical value^33^.

Though some mechanisms of control effects of synthetic jet on airfoil stall were revealed and recognized in current stage, there are some different conclusion on the control efffecs for incline angles of synthetic jet on delaying the stall of an airfoil. Several investigations indicated that a nearly tangent jet state, with the jet angle less than 30°, could enhance the aerodynamic performance of an airfoil more significantly^34–37^, whereas the comparison made by Monir et al. showed that an approximately 45° angle of a synthetic jet is the optimal one of NACA23012 airfoil in large angle of attack (AoA)^38^. Moreover, the numerical analyses by He et al. reported that the control effects of a synthetic jet were not sensitive to the jet angle^39^.

In this paper, to investigate the control effects of incline angle of synthetic jet and other parameters in mechanism, a series of wind-tunnel tests were conducted for parametric verifications of NACA0021 airfoil stall characteristics, including the measurement of velocity profile in boundary layer. In the studies the jet momentum coefficient, the jet angle and the jet location are incorporated thoroughly. In consideration of the inconsistent influences of the jet angle in actual tests, detailed investigations were carried out in a variety of freestream conditions and electrical excitation voltages to investigate the inconsistencies.

## Experiment and testing scheme

Considering the size of the synthetic jet actuators (SJA) and the installation of the actuator arrays in the wing (airfoil) model, the NACA0021 airfoil is used for the test model with a chord size *c* = 0.2 m and a span size *L*_*a*_ = 0.36 m. Because the cover plate of SJA is a flat surface, so the installation of the SJA changes the local geometry and curvature of original NACA0021 airfoil, Considering this imperfection, the SJA is installed at 0.15*c* and 0.4*c* respectively where the curvatures of the airfoil are relatively small. Though the test might obtian different data from that of a real NACA0021 airfoil, the experiment with and without SJA control are both based on the same model with the SJA installed, so the experiment could show the control effects of the SJA on flow separation and stall of airfoil.

**Figure 1:**
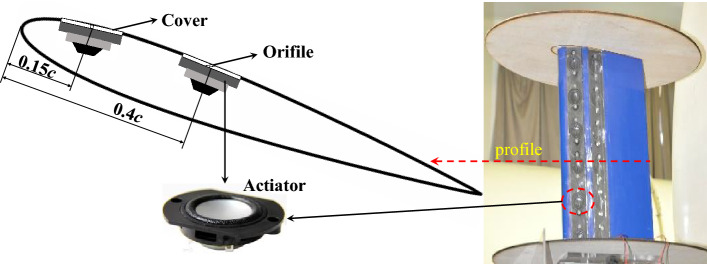
Pictorial configurations of the SJAs and their placements in the model.

The tests are conducted in the 0.75 m diameter working section closed-return wind tunnel of Nanjing University of Aeronautics and Astronautics^40^, with maximu wind speed 25 m/s and the turbulence intensity of the wind tunnel is about 1.2% measured by the hot wire anemometer with the model in the test section. The measurements are performed at chord-based Reynolds number *Re*_*c*_ ≈ 0.7 × 10^5^ ~ 3.5 × 10^5^ (freestream velocity V_∞_ = 5 ~ 25 m/s). The AoA of the airfoil is adjusted within a range of − 9° < *α* < 28° through a computer-controlled actuator. The wing (airfoil) model is mounted on a shrouded six-component force and moment balance system fixed on the tunnel test position. Two elliptical endplates are used and installed at both the ends of the wing model to avoid the influences of three-dimensional flow along the spanwise of wing model, as shown in Fig. 1, and the long and short axises of the endplate are 500 mm and 260 mm respectively.

The SJA is designed by the 1-inch full range speaker unit with the size being 43 mm × 35 mm × 16 mm, the resistance 4 Ω, impedance 8Ω, and the rated power 5 W. There are two slots arranged in the wing model at 0.15c and 0.4c respectively, and two cover plates (360 mm × 35 mm) are used to cover the slots. On each cover plate, the synthetic jet array is intalled with 6 actuators along the spanwise direction connected in parallel, as shown in the following figure. There are 6 orifices just on the actuators, and the length and width of the orifice are L_jet_ = 20 mm and h = 1.5 mm respectively, and the spacing of the two orifices on the same cover plate is D = 40 mm. The jet angle (θ_jet_ = 30°, 60° and 90°) is varied by changing the cover plate with different incline angle of orifice.

**Figure 2:**
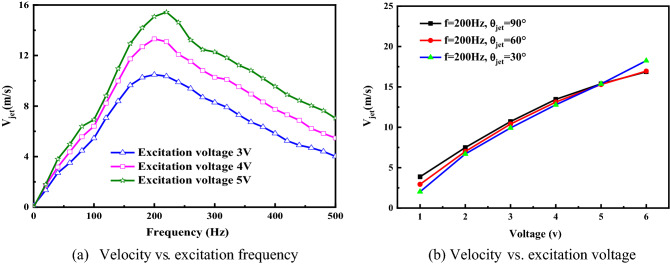
Jet mean velocity with different excitation frequency and voltage.

Figure 2 shows the mean velocity varies with the excitation frequency at the centerline of jet orifice of an isolated actuator according to different excitation voltage and different jet angle. As can be seen, the mean velocity of jet (*V*_*jet*_) increases with a larger excitation voltage (U_jet_), and there is a peak mean velocity when the excitation frequency (*f*_jet_) is about 200 Hz (which is adopted in the whole test). Therefore, the excitation frequency of each SJA is fixed 200 Hz (F^+^ = *f*_jet_ *c*/*V*_∞_ = 1.6 ~ 8) to ensure relative large jet mean velocity, and the jet velocity and momentum coefficient with different excitation voltage and freestream velocity are illustrated in Table 1.

**Table 1:** Jet mean velocity and momentum coefficient with different excitation voltage and freestream.

U_jet_	V_jet_ (m/s)	Kinetic efficiency^41^	Momentum coefficient $C_{\mu } = 2\frac{h}{c}(\frac{{V_{jet} }}{{V_{\infty } }})^{2}$			
			V_∞_ = 10 m/s	V_∞_ = 15 m/s	V_∞_ = 20 m/s	V_∞_ = 25 m/s
1 V	2.94	0.75%	0.0013	0.00057	0.00032	0.00021
2 V	7.07	2.60%	0.0075	0.0033	0.0019	0.0012
3 V	10.35	3.62%	0.016	0.0071	0.0040	0.0026
4 V	13.12	4.15%	0.026	0.011	0.0065	0.0041
5 V	15.35	4.25%	0.035	0.016	0.0088	0.0057

**Figure 3:**
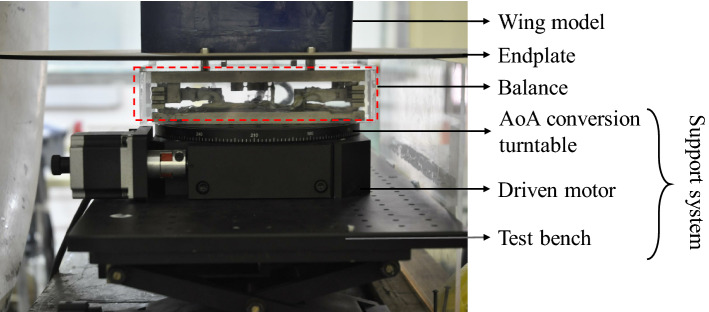
The balance and support system.

The wing model, balance and support system are show in the Fig. 3. The wing model is fixed in the central area of the wind tunnel test section by vertical installation. The wing model and the endplate is connected directly with the box balance through the standard mounting hole and installed on the support system. The support system contains angle of attack (AoA) conversion turntable, driven motor and test bench. The AoA control system contains the conversion turntable, driven motor, signal generator, and the control computer. When a required AoA of wing model is input into the computer, and the computer control the signal generater to send a signal and drive two-phase stepping motor, then the motor controls the worm gear mechanism to drive the AoA conversion turntable to rotate, so as to change the AoA(α) of the wing model. The control system can change the AoA of the wing model within ± 45°, and the minimum variation of AoA is 0.01°.

**Figure 4:**
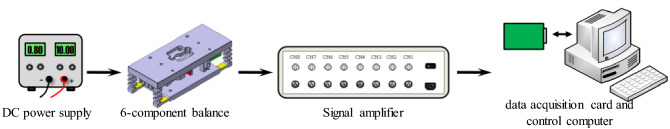
The force and moment measured system for six-component balance.

The aerodynamic forces and moment are measured by the six-component balance system as shown in Fig. 4, which contains the 6-component box aerodynamic balance, DC regulated power supply, signal amplifier with high precision, 16 bit data acquisition card, control computer and special test software. The measure range, accuracy and precision of the balance for different forces and moments are given in the Table 2.

**Table 2:** Static calibration performance of balance.

	Fx (Kg)	Fy (Kg)	Fz (Kg)	Mx (Kg.m)	My (Kg.m)	Mz (Kg.m)
Range	3	10	3	1	1	2
Accuracy (%)	0.307	0.432	0.400	0.298	0.458	0.299
Precision (%)	0.206	0.187	0.109	0.222	0.235	0.165

**Figure 5:**
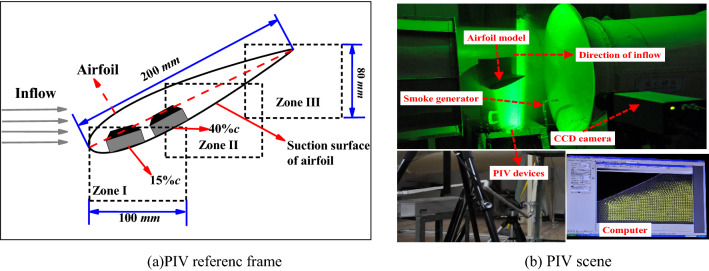
PIV measuring syetem of the test.

The velocity field over the airfoil is measured by PIV technology. The PIV system contains double pulse laser sources, optical element, CCD camera, synchronizing device, Insight 3G processing software and traditional solid particles devices etc. In order to improve the accuracy of the CCD camera for capturing the information of the local flowfield over airfoil, three valid zones of PIV measurement are distributed to comprehensively cover the suction surface of airfoil. The Insight 3G operating software for Windows is used to control the work of the entire PIV system. Firstly, a high-speed image acquisition card is used to capture the particle image of the flowfield from the CCD camera, and then the particle image is analyzed and processed by the cross-correlation algorithm to obtain the velocity vector of the whole flowfield. The main parameters of PIV system are given in Table 3. Figure 5 illustrates the model experimental equipment and PIV zones and measuring scene.

**Table 3:** Main parameters of PIV system.

Parameters	Value
Single pulse width (ns)	< 10
Maximum output power (mJ)	120
Pulsed laser wavelength (nm)	532
Pulse duration (ns)	10
Repeat Frequency of Double Pulse (Hz)	15
Pulse interval (μs)	0.5–33.3
Double frame frequency of CCD (Hz)	15
The resolution of CCD camera (Pixel )	1376 × 1024
Time interval of two frames (μs)	< 1
Valid measure-zone (mm)	100 × 80

**Figure 6:**
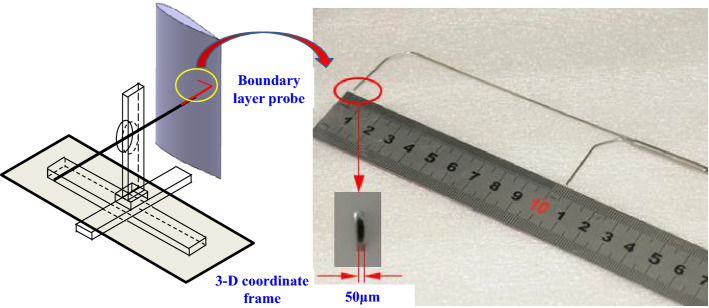
Schematic diagram of boundary layer probe.

Furthermore, the velocity profiles of boundary layer are evaluated by the boundary layer probe as shown in Fig. 6. The boundary layer probe test system consists of boundary layer probe (Preston tube), 8-Channel pressure transmitter, 16 bit A/D conversion data acquisition and post-processing software based on PC. The outer diameter of the boundary layer probe is only 50 μm. Therefore, the interference to the flow field is small.

## Parametric analyses

### The active flow control for airfoil stall

**Figure 7:**
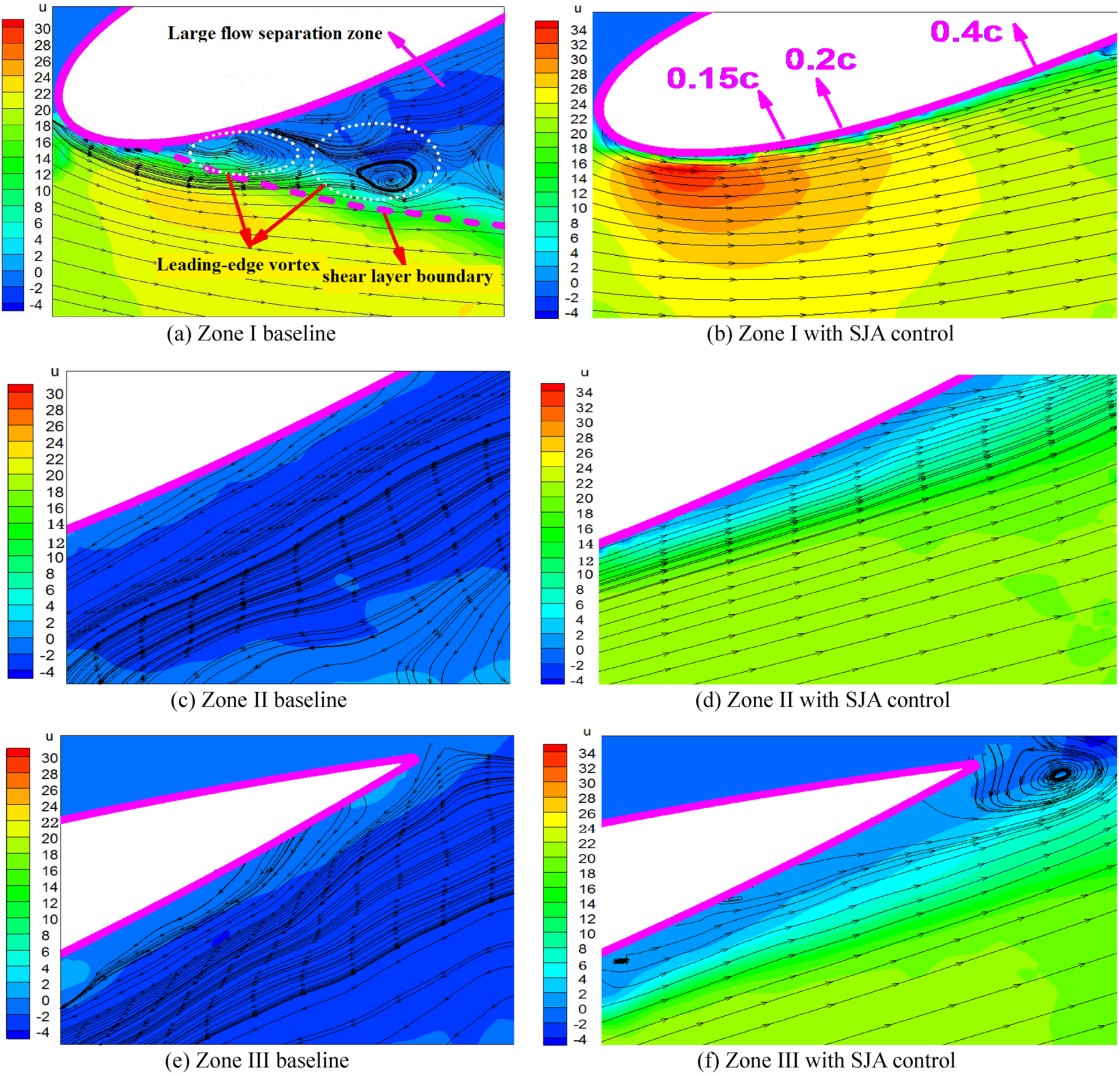
Velocity contours and streamline diagram of the airfoil under synthetic jet control.

The control effects of synthetic jets on preventing flow separations over airfoil are investigated at first, and Fig. 7 illustrates the velocity and streamlines of the three PIV zones (the PIV measurement plane is set on the center of the SJA orifice which is 150 mm away from the root of the wing installed on the support) over lower surface of the airfoil with and without jet control at *α* = − 21° under freestream velocity being 20 m/s. The 0.15*c* SJA is turned on at an angle of *θ*_*jet*_ = 90°. As shown, the stall vortex begins to form at the leading edge and its sequential convection along airfoil surface induced a large flow separation, resulting in a large-scale recirculation region near the trailing edge position containing zone II and zone III. The large flow separation finally causes a loss of lift, which threatens the efficiency of the airfoil. With the control of leading-edge synthetic jet, the mixing between the internal layer and the external layer of the boundary layer is heightened, and the energy of the entire boundary layer is strengthened consequently. Above effects enabled the effective inhibition of the leading-edge vortex and further makes the separation point move downstream along the airfoil, thus it could reduce the large flow separation region significantly which is illustrated in the figure.

### Control effects of synthetic jet on boundary layer

The effects of synthetic jet on separation flow are caused by its interaction on boundary layer, so in order to investigate the control mechanism of synthetic jet on boundary layer in post stall condition, the velocity profiles in boundary layer at 0.2*c* and 0.4*c* are measured by boundary layer probe.

**Figure 8:**
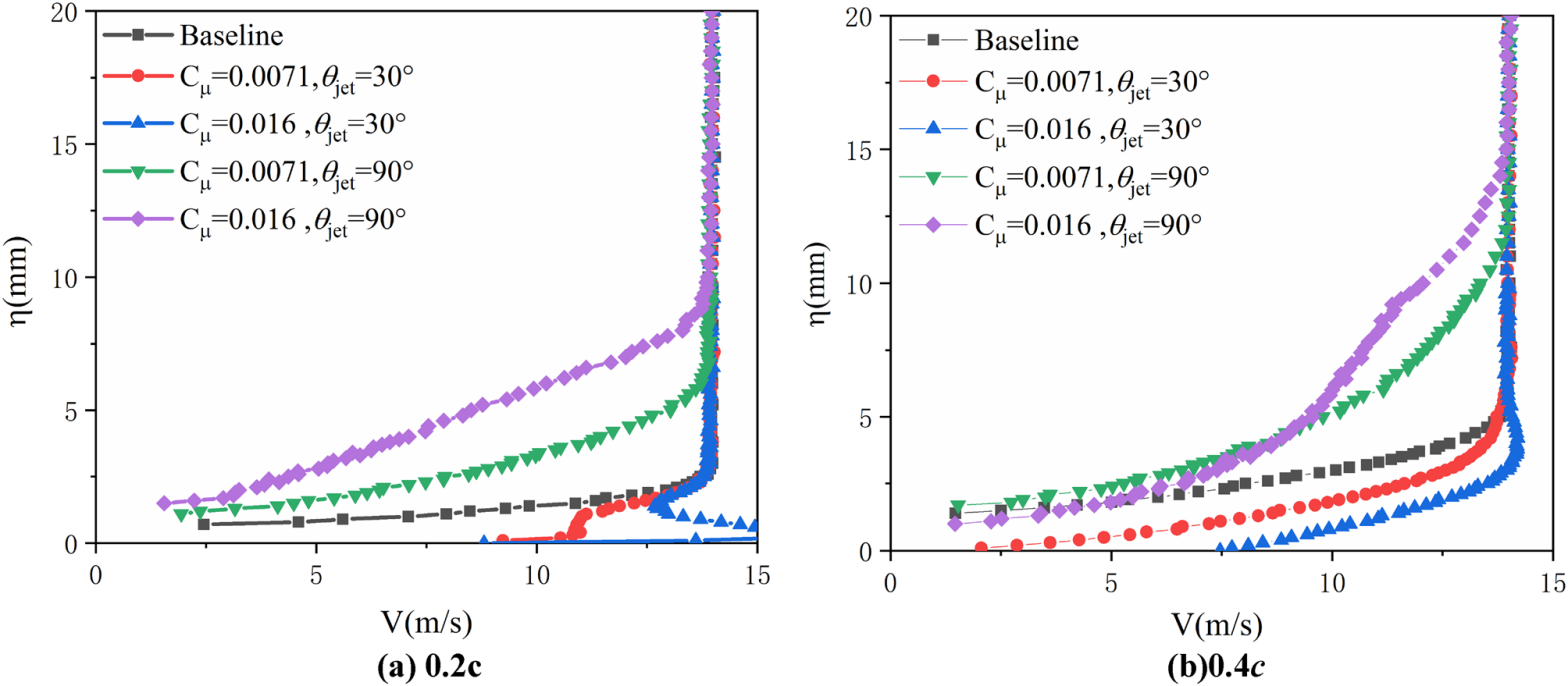
Boundary layer velocity profiles at *V*_∞_ = 15 m/s, *α* = 20°.

Figure 8 shows the results of the velocity profile measurements with different excitation voltages and jet angle of actuator 1 when freestream velocity is 15m/s and AoA=20°. As can be seen, and the synthetic jet upstream could change the velocity profile in boundary layer. When jet angle is small, the control of synthetic jet could hardly change the flow pattern and the thickness of the boundary layer, but the jet might inject energy and enlarge the velocity of the flow in boundary which helps to inhibited flow separation, resulting in the reattachment of the airflow over airfoil and delaying of airfoil stall. Additionally, as can be seen in Fig. 8a, the jet with 30° incline angle could accelerate the flow in inner boundary layer even larger than the freestream velocity, and the thickness of the boundary layer is almost unchanged. Differently, the jet with 90° angle (normal to the airfoil surface) could significantly change the flow characteristics of boundary layer, and with synthetic jet control the velocity profile gradually enlarge the thickness of the boundary layer. Besides, the thickness of boundary layer enlarges with the increment of jet angle and orifice velocity (momentum coefficient), and the thickness of boundary layer at 0.4*c* is larger than at 0.2*c*. Though synthetic jet with 90° angle might not inject energy to the boundary layer directly, it could enhance the mixture of flow in and out of boundary layer, and enlarge the thickness of boundary layer and further make the flow hard to separate from the surface of airfoil.

**Figure 9:**
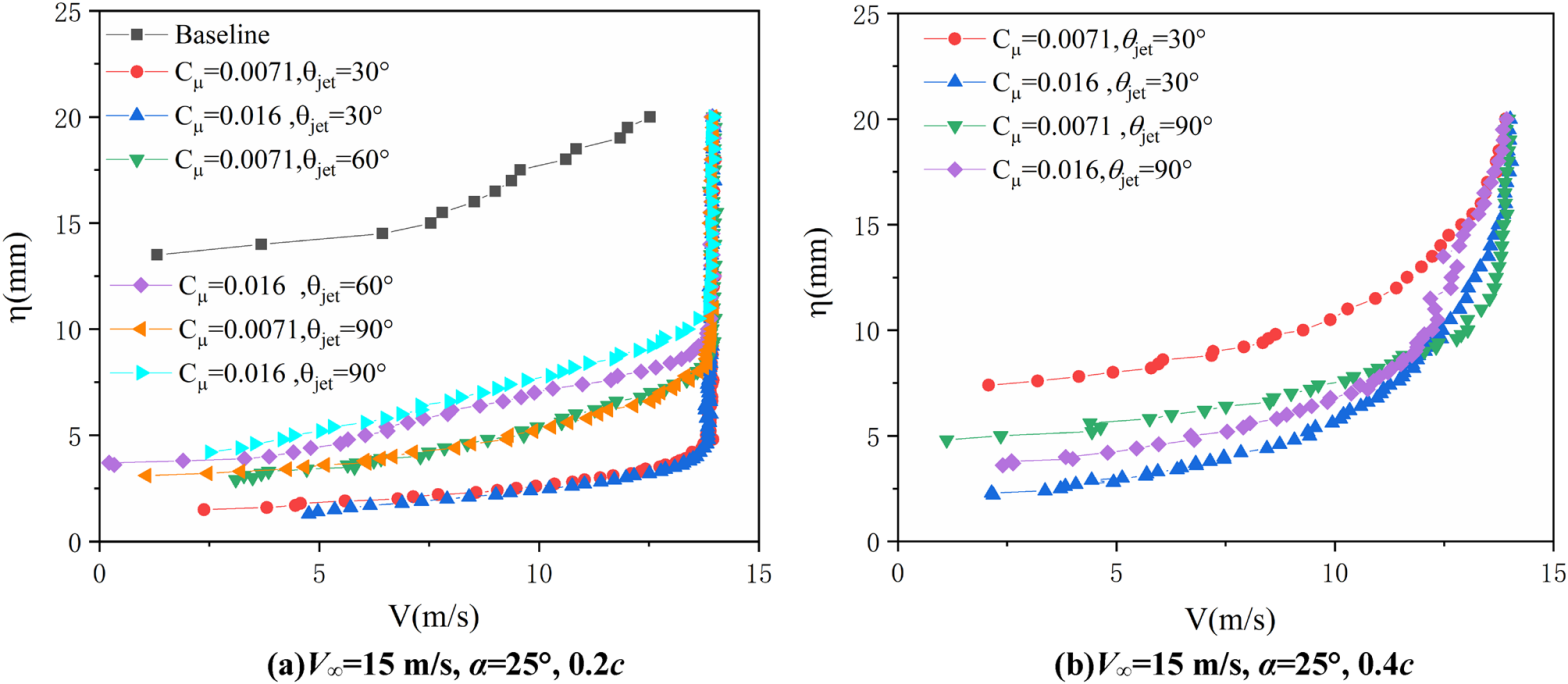
Test results of the boundary layer velocity profiles (*V*_∞_ = 15 m/s, *α* = 25°).

Figure 9 illustrates the velocity profile when freestream velocity is 15 m/s and AoA = 25°. It should be noted that the boundary layer probe cannot measure negative velocities, so the chord position is in recirculation zone if there is no velocity profile measured, baseline in Fig. 9b for example. As shown in the figure, if there is no jet control, the results at 0.2*c* and 0.4*c* are demonstrated to be large recirculation zone, especially apparent at 0.4*c.* Once jet control is turned on, flow separation is well suppressed, and the separated flow reattaches to the surface of the airfoil, and the effects of jet angle are similar to the case of airfoil with 20° angle of attack. Besides, comparing to the jet with 60° and 90° incline angle, the jet with 30° angle incline to the airfoil surface has a better control effect on control the separation flow of inner boundary. It is because that the nearly tangential jet could inject energy to the boundary layer directly, which could suppress the flow separation due to the adverse pressure gradient.

**Figure 10:**
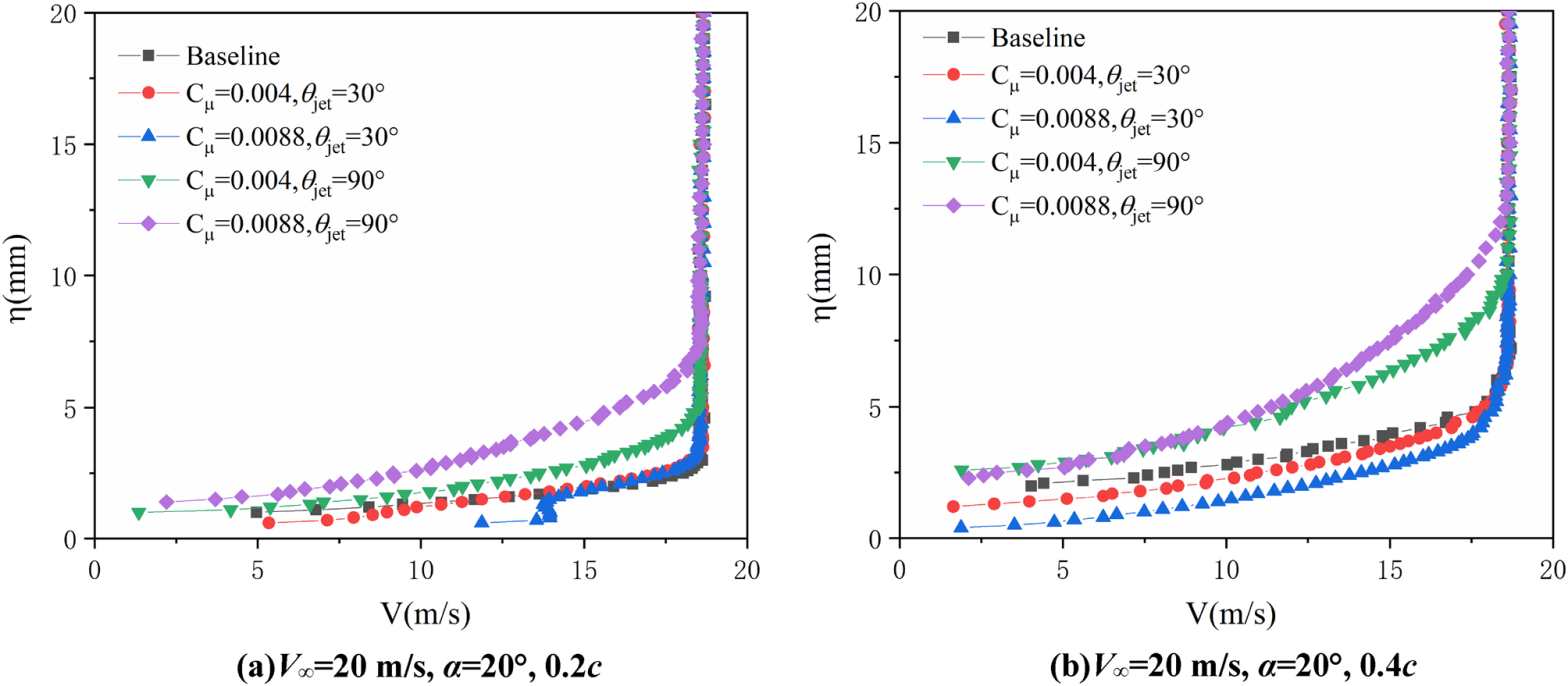
Test results of the boundary layer velocity profiles (*V*_∞_ = 20 m/s, *α* = 20°).

**Figure 11:**
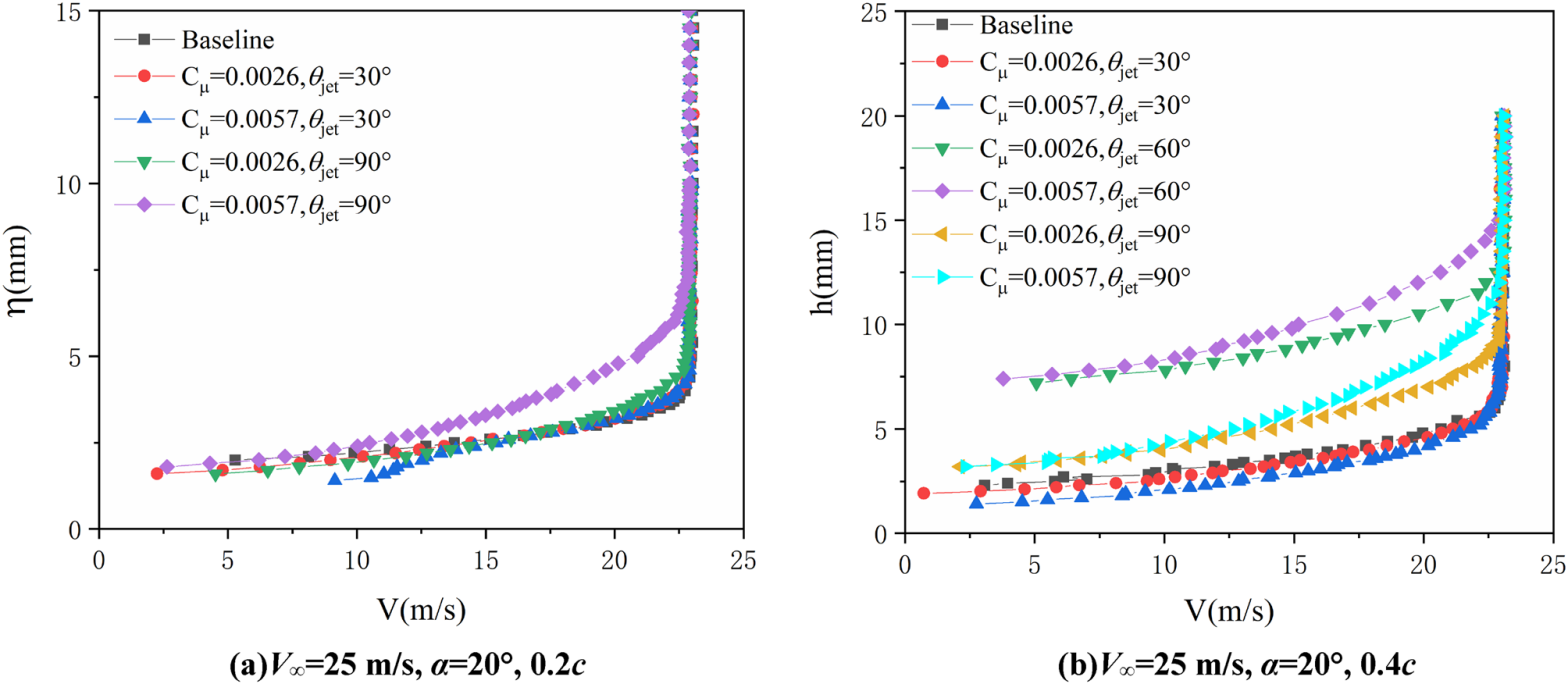
Test results of the boundary layer velocity profiles (*V*_∞_ = 25 m/s, *α* = 20°).

**Figure 12:**
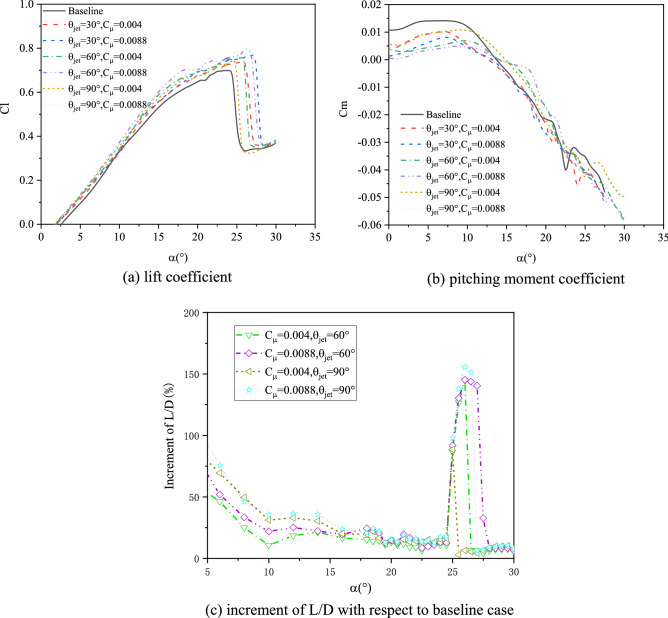
Test results of the aerodynamic forces (*V*_∞_ = 20 m/s).

Figures 10 and 11 give the velocity profile in boundary layer at 0.2*c* and 0.4*c* on suction surface of airfoil with AoA = 20° when freestream velocity is 20 m/s and 25 m/s respectively. The control effects of synthetic jet are similar to the condition when freestream velocity is 15 m/s. In order to investigate the relation between flow characteristics in boundary layer and aerodynamic force of airfoil, the relevant lift, lift-to-drag ratio and pitching moment coefficients are presented in Fig. 12, the AoA step is 0.5°in the test. As can be seen, with synthetic jet control the lift coefficient increase at all of the AoA state, and stall is significantly delayed which is caused by the inhibiting of flow separation with jet control.

### Effects of the jet location

**Figure 13:**
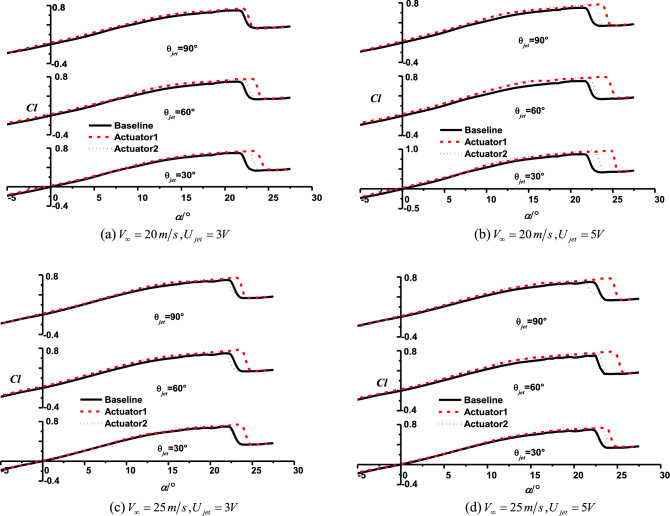
Comparison of stall control effect between A1 and A2.

The investigation on control effects of SJA with different locations are carried out in this part. Figure 13 shows the contrastive control effects of the two SJAs (the one placed at 0.15c is named A1 and the other one at 0.4c is named A2) with different freestream velocity (V_∞_ = 20 m/s and V_∞_ = 25 m/s) and varied jet parameters (θ_jet_ and C_μ_). As shown in the figure, at all the freestream velocity, A1 has better control effects than A2 on increasing the maximum lift coefficient and delaying stall AoA of airfoil when the two SJAS have the same jet angles and momentum coefficients. The reason is that stall of airfoil is induced by the large flow separation near the leading edge, and A1 is closer to the separation point, so the synthetic jet could inject momentum to the boundary layer and enhance the mixing of the shear layer more directly. Moreover, when the magnitude of inflow was relatively large, the SJA2 with a 60° or 90° jet angle might lead to the decrease of stall AoA.

### Effects of the jet angle

**Figure 14:**
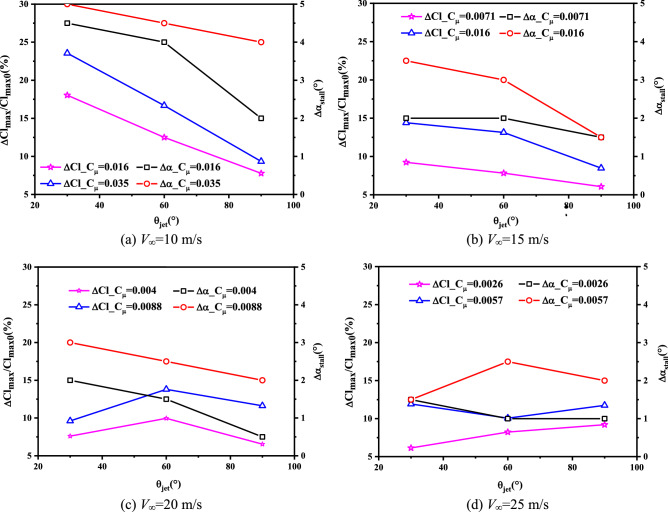
Effects of jet angles and momentum coefficients to the stall characteristics of airfoil.

The disagreements about the influences of jet angle on synthetic jet controls exist for many years, it is necessary to find out the reasons or promote further relevant investigation by means of experimental technologies. In this part, a series of freestream conditions (10 ~ 25 m/s) are considered to carry out the experimentally parametric analyses. Figure 14 shows the relationships between the increments of stall angle and the maximum lift coefficient of airfoil under SJA1 control, with the jet driven by varied excitation voltages and jet angles under different freestream conditions.

As shown in the figure, when the freestream velocity is lower than 15 m/s, the control effects is demonstrated to be wakened as the value of jet angle increases, which is in accordance with Hassan’s results^36^. As the freestream velocity increases (20 m/s, for example), the control effects on increasing the lift coefficients and the stall angles of airfoil decrease. The reason might be that the decrease in jet momentum coefficient suppress the energization from synthetic jet, and thus the control effects are less impacted by the jet angle (especially in the case when the voltage being 3 V). The phenomenon is also similar to the numerical results of He^38^.

Overall, for the freestream with relatively larger velocities, the control mechanisms of SJA from the jet angle are a little different from the cases under smaller freestream velocities. The interactions induced by synthetic jet to the flowfield over airfoil are mainly due to the mixing effects when the momentum coefficient of a jet becomes much smaller, and a jet with a larger inclined angle (nearly normal jet) could penetrat the farthest into the freestream thus enhance the mixing effects between inner layer and outer boundary layers, which has been proved by Buren^9^. As shown in Fig. 14, the jet with 60° inclined angle has the best control effect on increasing the lift coefficient under an freestream velocity being 20 m/s, while the jet with 90° jet angle is superior on the lift improvement under 25 m/s freestream, it is because the wall-normal jet induced the most flow unsteadiness near the orifice^9^, which could delay the flow separation.

The experimental results indicate that the influences of the jet angle on the stall control of airfoil by synthetic jet are very complicated, and only considering a single jet angle could not obtain the best control effects for all freestream and excitation states, and such effects are highly dependent on the jet momentum coefficient. Better control effects could be achieved by setting jet to a smaller angle when the momentum coefficient is large enough. The difference is, when the jet momentum coefficient is small to a reference extent, a larger jet angle is required to improve the control effects on delaying the stall of airfoil.

### Effects of jet velocity

The mean velocity is an important parameter for synthetic jet. To investigate its influences on airfoil stall and flow separation, four excitation voltages (2 ~ 5 V, with resulting jet velocity about 7 ~ 16 m/s) are conducted to explore the effects of velocity of synthetic jet on enhancing the aerodynamic characteristics of a stalled airfoil.

**Figure 15:**
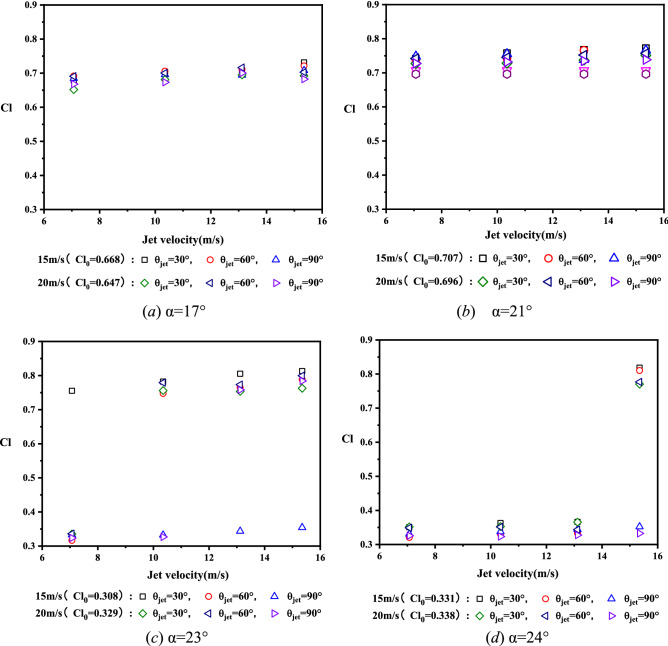
Lift coefficients of airfoil with and without jet control at different AoAs.

Figure 15 gives the lift coefficients of airfoil under control of SJA1 at different jet angles, AoA and freestream velocity. For the conditions with the same freestream velocity, the increment of jet velocity leads to the enlargement of jet momentum coefficient, as a result, the control effects of synthetic jet on airfoil lift coefficient are enhanced. It is obvious that a jet with larger velocity enhanced the interactions between the periodic synthetic jet and the flow in boundary layer, and it could also strengthen the mixing of the inner and outer layers in the boundary and resulted in the increment of airfoil lift. However, the control efffects of synthetic jet weankens at much larger AoA under post stall, it is because that the separation point moved toward the leading-edge of airfoil, and the jet in the recirculation zone with small velocity has not enough energy to control the large flow separation.

### Effects of the freestream condition

As another parameter affects the momentum coefficient of synthetic jet, the control effects of synthetic jet with different freestream velocity are investigated, and the comparisons about airfoil lift coefficients versus AoA are conducted at different freestream velocity.

**Figure 16:**
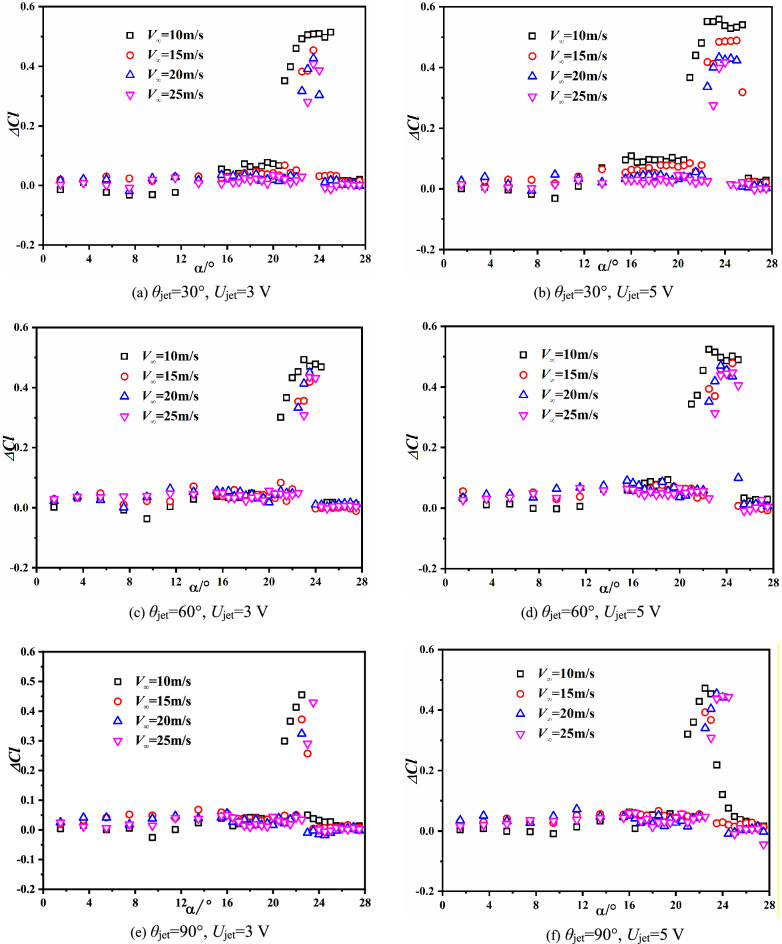
Variations of lift coefficient with different momentum coefficient.

Figure 16 shows the variations of lift coefficients under SJA1’s control with different freestream velocity. The jet momentum coefficient decreases with the increasing of freestream velocity, and the reduction of jet momentum coefficient weakens the SJA control effects on increment of stall angle and lift of airfoil.

The comparison about results also reveal the mechanisms of synthetic jet control about the jet angle. As shown in the plots, the control effects in smaller incline angle are more effective with respect to various freestream conditions, indicating that the control effects in nearly tangent jet would be significantly impacted by the jet momentum. Unlike the small incline angle cases, a normal jet configuration could control the aerodynamic characteristics of airfoil with less implication of the freestream velocity (jet momentum).

**Figure 17:**
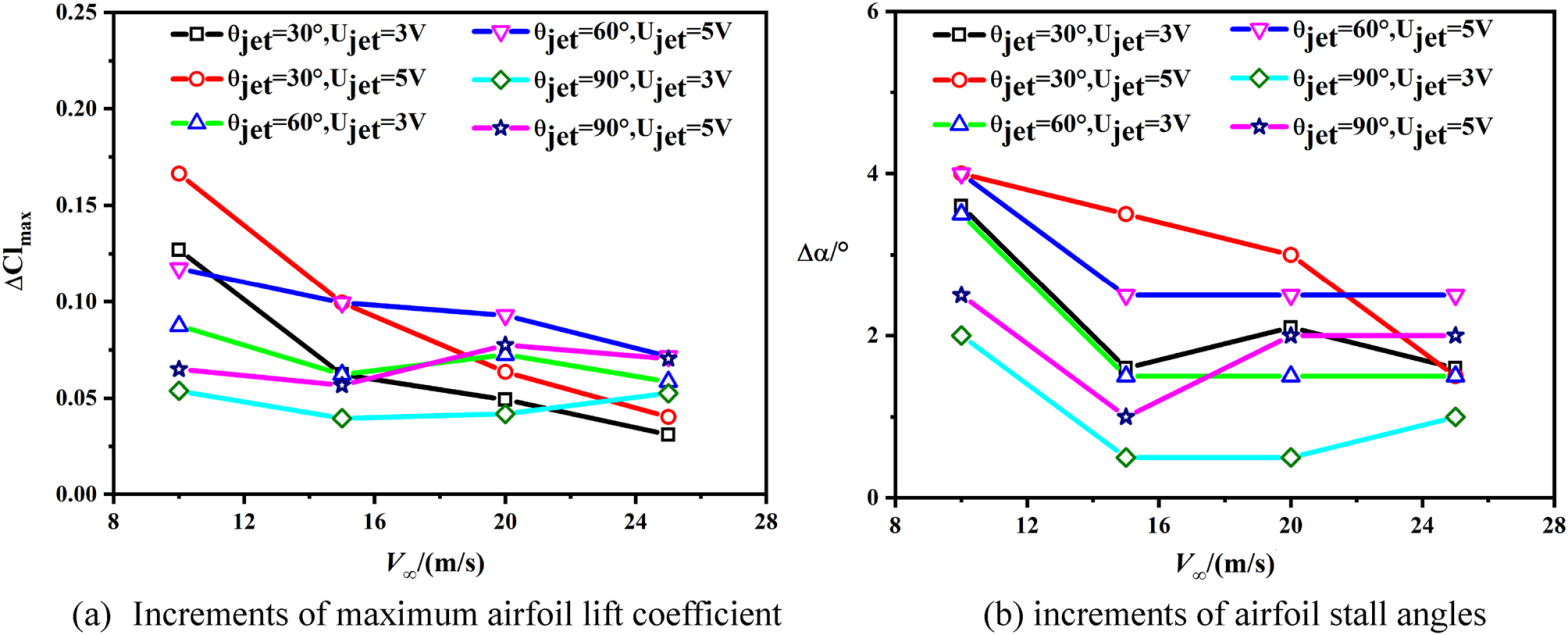
Influences with an increment of stall-incidence under different momentum coefficient.

To expound the control effects of synthetic jet on delaying the stall of airfoil more visually, Fig. 17 illustrates the increments of airfoil stall angle and maximum lift coefficient with varied freestream velocity. With a low freestream velocity, the stall angle increases with the jet velocity. With the increasing of the freestream velocity, the control effects of the jet reduce. Note that, when the freestream is larger than 20 m/s (also larger than the maximum jet velocity), the control effects in the normal jet case might be improved. This adverse trend phenomenon is mainly due to the mixing effect aroused by the normal jet, because this effect could translate the outer-layer flow with high momentum to the inner layer boundary with low momentum. Therefore, the normal jet might have potentials to improve the stall angle of an airfoil with large freestream velocity.

## Conclusion

In this paper, the experimental investigations regarding the control effects of synthetic jets on the lift and stall angle of airfoil have been done, the tests contain the measurements of flowfield over suction surface of airfoil, the velocity profiles in the boundary layer and the overall aerodynamic forces of the model. Based on the comprehensive parametric analyses, the disagreements of synthetic jet control mechanisms among different references are partly explained by working out the coupling effects of the jet parameters. The most specific conclusions are shown as following:

1. The control effects of synthetic jets on the stall characteristics of airfoil could be effectively evaluated in both micro and macro ways through the measurements of aerodynamic forces, the transient flowfield using PIV technology and the velocity profiles of boundary layer.
2. The tangential jet inject energy and accelerate the velocity of flow in inner boundary layer, which helps to suppress the flow separation and delay airfoil stall. The normal jet enhances the mixing of flows in inner and outer boundary layer, which helps to enlarge the thickness of boundary layer and reduce the drag coefficient of airfoil when the momentum coefficient of synthetic jet is relatively small.
3. Arranging the SJA near separation point over airfoil could suppress the flow separation more effectively. Overall, a jet with larger momentum coefficient has better control effects on improving the lift coefficient of an airfoil and also increasing the stall angle.
4. In view of the previous disagreements about the control effects of the jet angle, the present investigations indicate that the control effects of jet angle are sensitive to the freestream conditions and the jet momentum coefficient. When the momentum coefficient of a jet is relatively larger (the jet velocity is as large as the freestream velocity), small jet angle tends to have better control effects on delaying the stall and improving the lift coefficient of an airfoil. When jet coefficient become relatively small, larger jet angle state is demonstrated to be more expectant to obtain optimal control.

## Data Availability

The datasets used and/or analysed during the current study available from the corresponding author on reasonable request.

## List of symbols

*a*: Local speed of sound propagation
*b*: Half width of synthetic jet
*c*: Chord length of wing model
*Cd*: Drag coefficient of airfoil
*Cl*: Lift coefficient of airfoil
*C*_*lmax*_: Maximum lift coefficient of airfoil
*Cm*: Pitching moment coefficient of airfoil
*C*_*μ*_: Momentum coefficient of synthetic jet
*D*: Spacing of two orifices
*f*: Excitation frequency of synthetic jet
*F*^+^: Non-dimensional frequency of synthetic jet
*h*: Width of jet orifice
*L*_*a*_: Span size of wing model
*L*_*jet*_: Length of jet orifice
Rec: Chord-based Renolds number
*U*_*jet*_: Excitation voltage
*V*_∞_: Freestream velocity of the wind-tunnel
*V*_*jet*_: Mean velocity of synthetic jet
*α*: Angle of attack, positive in nose-up motion
*Δα*_*stall*_: Increment of angle of attack
*ΔCl*: Increment of lift coefficient
*ΔC*_*lmax*_: Increment of maximum lift coefficient
*θ*_*jet*_: Jet angle of synthetic jet
